# Global Potential Distribution and Climatic Niche Dynamics of the Invasive Alien Weed *Solanum rostratum* Dunal

**DOI:** 10.1002/ece3.74662

**Published:** 2026-11-09

**Authors:** Yabin Liu, Weitian Meng, Lizhu Guo, Yuyu Li, Rui Wang, Chuan Wang, Yu Ji, Tian Tian, Lifen Hao, Kejian Lin

**Affiliations:** ^1^ Key Laboratory of Biohazard Monitoring and Green Prevention and Control for Artificial Grassland, Ministry of Agriculture and Rural Affairs Grassland Research Institute, Chinese Academy of Agricultural Science Hohhot China; ^2^ School of Plant Protection Anhui Agricultural University Hefei China; ^3^ State Key Laboratory for Biology of Plant Diseases and Insect Pests Institute of Plant Protection, Chinese Academy of Agricultural Sciences Beijing China

**Keywords:** biological invasion, climatic niche dynamics, COUE framework, invasion risk assessment, *Solanum rostratum*

## Abstract

Whether invasive species conserve their ancestral climatic niches or undergo realized niche changes during global spread fundamentally affects the reliability of invasion risk projections. We integrated ensemble species distribution models (SDMs), the extended COUE framework, and Multivariate Environmental Similarity Surfaces (MESS) to quantify the global climatic niche dynamics and invasion risk of *Solanum rostratum* Dunal (Solanaceae) across its native North America and four invaded regions. Macroecological modeling revealed a temperature‐driven, precipitation‐constrained distribution, where thermal availability and mean diurnal range dictated suitability, while precipitation extremes imposed strict limits. Cross‐regional analyses comparing true occurrence records revealed a highly heterogeneous continuum of realized niche dynamics. Populations in Australia and Russia–Eastern Europe exhibited strict climatic conservatism, occupying analogous climates with complete niche stability (S = 1.000). Western European populations displayed substantial niche unfilling (U = 0.321); however, 80.4% of this constituted abandonment (native climates absent from Europe), suggesting genuine dispersal limitation. In contrast, Chinese populations exhibited extensive colonization of novel climatic conditions. Although showing near‐complete stability strictly within available analogous climates (S = 0.998), 48.5% of the expansion‐related niche quantity in China occurred in non‐analog environments (pioneering), leading to poor projection transferability of the native SDM (Continuous Boyce Index = 0.166). These findings demonstrate that *S. rostratum* maintains ancestral climatic preferences where suitable environments are available while possessing the capacity to pioneer novel climatic spaces during global invasion. Integrating these expanded realized tolerances into a global ensemble SDM provides a robust, region‐specific risk assessment framework that improves projection accuracy and supports adaptive management under global environmental change.

## Abstract 2

By integrating ensemble species distribution models with niche dynamic frameworks, we investigated the global invasion of *Solanum rostratum* to resolve whether invasive species retain ancestral climatic niches or undergo adaptive shifts. Our analyses reveal a region‐specific continuum ranging from strict niche conservatism in Australia to substantial adaptive niche expansion into colder and drier environments in China. These findings demonstrate that relying solely on native‐range data systematically underestimates invasion potential, highlighting the necessity for dynamic, region‐specific risk assessment frameworks in global biosecurity management.

## 1 Introduction

Biological invasions are among the major drivers of global biodiversity loss, with far‐reaching consequences for agricultural productivity, ecosystem resilience, and socio‐economic stability (Jaureguiberry et al. 2022; Turbelin et al. 2023). Intensifying global trade and transportation have accelerated human‐mediated species dispersal across biogeographic boundaries (Early et al. 2016; Hulme 2009, 2021). Meanwhile, climate change is reshaping temperature and precipitation regimes, potentially allowing alien species to overcome historical environmental constraints and establish populations in previously unsuitable regions (Deslippe and Veenendaal 2025; Hellmann et al. 2008). Therefore, accurate assessments of invasion potential require not only identifying currently suitable climatic conditions but also understanding whether introduced populations retain ancestral environmental preferences or develop novel ecological tolerances (Flickinger and Dukes 2024; Rubenstein et al. 2023; Wallingford et al. 2020).

Species distribution models (SDMs) are widely employed in invasion biology to estimate potential distributions based on species occurrences and environmental predictors (Elith et al. 2010). However, projections beyond the calibration region commonly rely on the assumption of climatic niche conservatism—the premise that invasive populations maintain the climatic relationships established within their native range (Petitpierre et al. 2012). When this assumption is violated, models calibrated solely with native‐range data systematically mischaracterize invasion risk, potentially underestimating the threat in regions where introduced populations undergo realized niche changes through mechanisms including phenotypic plasticity, enemy release, or genetic differentiation (Atwater et al. 2018; Biancolini et al. 2026; Dlugosch and Parker 2008; Liu, Wolter, et al. 2022).

Quantifying changes in the realized niche between native and invaded ranges is therefore essential for both testing model transferability and understanding environmental colonization strategies (Broennimann et al. 2012). The Centroid Shift, Overlap, Unfilling, and Expansion (COUE) framework provides a rigorous method to decompose realized niche dynamics into distinct components: niche stability, expansion, and unfilling (Guisan et al. 2014). Recent advancements in this approach further distinguish true niche divergence from differences in environmental availability by identifying “pioneering” (expansion into non‐analog novel climates) and “abandonment” (unfilling caused by the absence of native climates in the invaded region) (Broennimann et al. 2026). Combined with Multivariate Environmental Similarity Surfaces (MESS), these approaches explicitly disentangle whether introduced populations occupy climates analogous to their native range or colonize entirely novel environments (Di Cola et al. 2017; Melton et al. 2022).

*Solanum rostratum* Dunal (Solanaceae), commonly known as buffalobur, is a widely established invasive annual herb native to North America. It has successfully established populations across climatically contrasting regions, including China, Australia, Russia–Eastern Europe, and Western Europe, and is considered a high‐risk alien species in many countries (Yu et al. 2021; Zhao et al. 2013). It produces toxic alkaloids such as solanine, posing risks to livestock and reducing rangeland productivity (Lin and Tan 2007). Furthermore, it acts as an intermediate host for *Leptinotarsa decemlineata* and multiple plant viruses, creating substantial threats to agricultural biosecurity (Izzo et al. 2014; Matzrafi et al. 2023).

Existing studies on *S. rostratum* have primarily focused on regional occurrence mapping or local trait variation (Huang, Yang, et al. 2024; Wang et al. 2025). However, whether populations established across different continents share similar realized climatic requirements or exhibit region‐specific niche dynamics remains unresolved. The stark climatic contrasts between invaded regions—such as the arid continental environments of northern China versus the cooler, wetter environments of Western Europe—provide a unique opportunity to test whether this species follows strict climatic conservatism or exhibits broader realized climatic tolerances globally.

Here, we investigated the global climatic niche dynamics and invasion risk of *S. rostratum* by integrating native‐range and global SDMs with empirical environmental‐space analyses. Specifically, our objectives were to: (1) evaluate the transferability of a native‐calibrated SDM across invaded regions to directly test realized niche conservatism; (2) quantify regional differences in climatic niche stability, expansion, unfilling, pioneering, and abandonment using updated COUE and MESS approaches; (3) identify the dominant climatic drivers underlying global suitability patterns; and (4) develop a global ensemble SDM incorporating the species' complete realized climatic tolerances to improve spatial risk mapping.

## 2 Materials and Methods

### 2.1 Occurrence Data Sourcing, Processing, and Spatial Thinning

Global occurrence records of *S. rostratum* were comprehensively compiled from multiple biodiversity databases and field surveys. These sources included the GBIF (accessed May 2025), iNaturalist, CABI, CalFlora, the Atlas of Living Australia, the Southwest Environmental Information Network (SEINet), and targeted field surveys conducted in China.

To ensure consistency between occurrence records and contemporary climatic conditions represented by the environmental predictors (1970–2000), we retained only recent records representing established populations. Following current best practices for species distribution modeling, historical herbarium and museum specimen records (prior to 1970) were excluded because they often contain substantial temporal sampling biases and may not accurately represent current population distributions (Christina et al. 2020).

Data extraction and initial filtering were conducted in R. Only records with valid geographic coordinates and reliable occurrence information were retained. To adhere to contemporary reporting and reproducibility standards, spatial errors were explicitly identified and removed using the *CoordinateCleaner* package (Feijó et al. 2021; Zizka et al. 2019). Specifically, common coordinate errors including country or province centroids, institutional coordinates, zero coordinates, and ocean coordinates were assessed and eliminated. Records with coordinate uncertainty greater than 100 km were also removed.

After strict quality control, occurrence records from GBIF were combined with those obtained from other biodiversity databases and field surveys. The initial dataset contained more than 26,000 records worldwide. Duplicate records with identical geographic coordinates were manually identified and removed, resulting in 25,423 unique occurrence records. Because biodiversity databases often contain spatially clustered observations associated with uneven opportunistic sampling effort, spatial rarefaction was subsequently performed using the *spThin* package to reduce spatial autocorrelation and directional sampling bias (Aiello‐Lammens et al. 2015). A minimum distance threshold of 10 km was applied, approximately corresponding to the spatial resolution of the environmental predictors (~10 km at the equator), allowing only one occurrence record to be retained within each grid cell. After spatial thinning, the final high‐quality dataset consisted of 4579 globally distributed occurrence records, which were used for subsequent modeling and climatic niche analyses.

### 2.2 Climatic Predictor Selection and Ecological Justification

To represent global climatic dimensions, 19 bioclimatic variables (1970–2000) at a 5‐arc‐minute resolution (~10 km) were retrieved from WorldClim v2.1. To address multicollinearity without arbitrarily discarding biologically relevant predictors, we implemented a two‐stage selection approach combining ecological prior knowledge with combinatorial statistical testing.

First, we identified highly correlated variable pairs (Pearson's |*r*| > 0.7) across the global occurrence grid cells. Rather than arbitrarily dropping variables based purely on correlation matrices, we constructed 12 alternative, low‐collinearity variable combinations designed to capture distinct thermal and moisture dimensions relevant to the species' annual life history (Table S1). Second, we conducted a preliminary variable screening experiment using a baseline ensemble SDM to evaluate the model performance of these 12 combinations (Table S2). Combination 12 consistently achieved the highest predictive accuracy (AUC = 0.976, TSS = 0.844) and was thus selected as the optimal predictor set.

Crucially, in accordance with robust modeling protocols (Mancini et al. 2025), the final selection of Combination 12 was not driven solely by statistical performance; it is fundamentally ecologically justified as it captures the critical physiological constraints of *S. rostratum* . This final set included nine bioclimatic variables: annual mean temperature (bio1), mean diurnal range (bio2), temperature annual range (bio7), mean temperature of the wettest quarter (bio8), mean temperature of the warmest quarter (bio10), precipitation of the wettest month (bio13), precipitation of the driest quarter (bio17), precipitation of the warmest quarter (bio18), and precipitation of the coldest quarter (bio19). Specifically, bio1, bio2, and bio7 define the species' broad thermal tolerance boundaries and adaptation to continental temperature fluctuations. Bio8 and bio10 dictate the thermal energy available during the critical active growing and summer flowering phases. Moisture constraints are well represented by bio13 and bio17. Finally, bio18 and bio19 capture the essential seasonal moisture‐temperature synchrony governing its life cycle.

### 2.3 Delineation of Focal Study Regions and Accessible Areas (M)

Occurrences were grouped into one native region (North America: *n* = 3871) and four focal invaded regions: China (*n* = 434), Western Europe (*n* = 126), Russia–Eastern Europe (*n* = 76), and Australia (*n* = 31). Occurrences falling outside these five focal regions were excluded from regional niche comparisons but were retained for calibrating the final global SDM. To avoid the methodological flaws of using arbitrary rectangular extents, which lack ecological justification and artificially inflate model discrimination, we strictly defined the species' accessible areas (M spaces) using biologically meaningful boundaries (Rojas‐Soto et al. 2024). For each specific focal region, as well as for the entire global dataset (all 4579 records), we spatially intersected the true occurrences with the WWF Terrestrial Ecoregions of the World (TEOW) shapefile (Olson et al. 2001). All ecoregion polygons containing at least one occurrence were extracted and dissolved to form continuous, ecologically realistic accessible areas. These explicitly defined M areas were used to constrain both SDM background sampling and environmental‐space niche quantifications (Figure 1).

**FIGURE 1:**
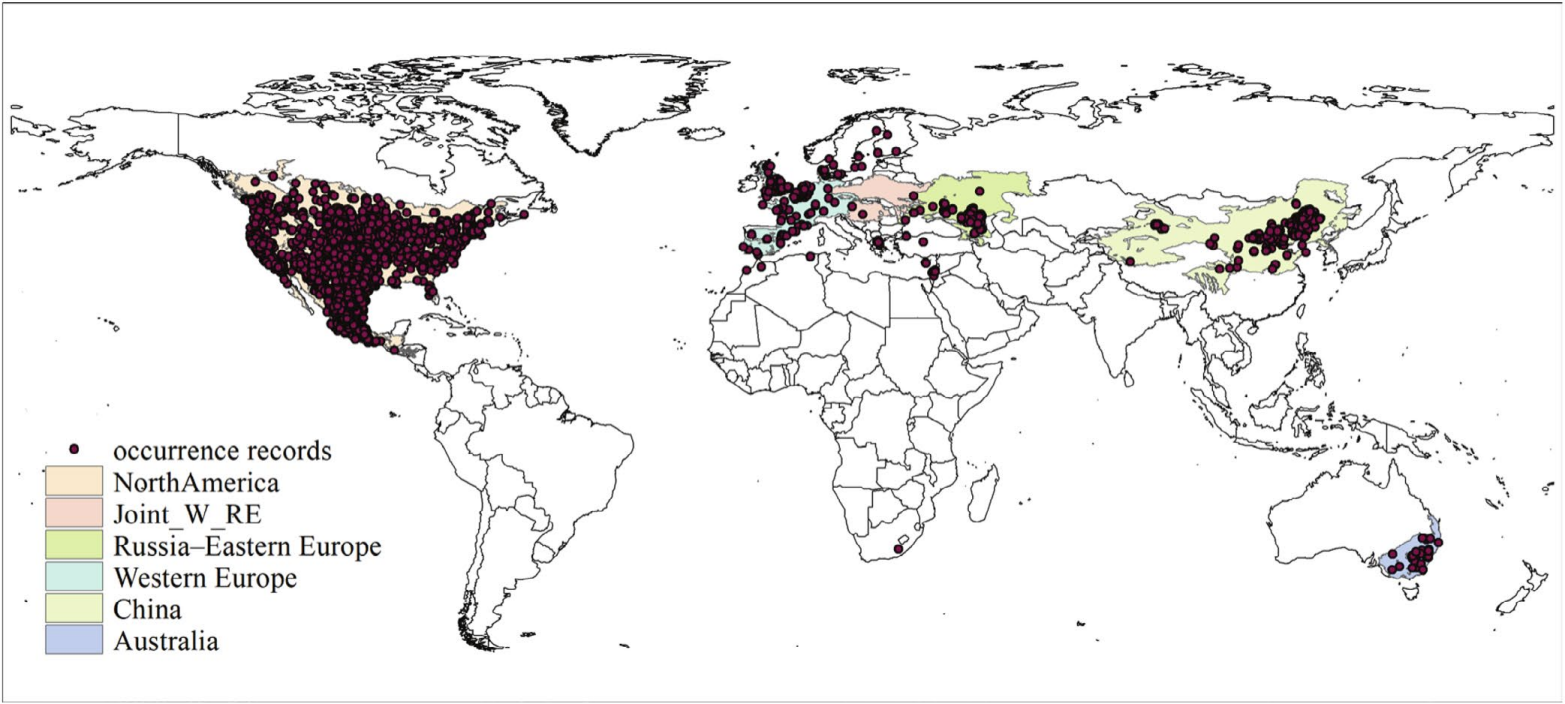
Global occurrence records and accessible areas (M) used for climatic niche analyses of *S. rostratum* .

### 2.4 Species Distribution Modeling and Spatial Transferability

To project the potential distribution of *S. rostratum* , we employed an ensemble modeling approach using the *biomod2* package in R. Based on current best practices, we excluded the outdated SRE algorithm and retained robust algorithms, including GLM, MARS, GAM, CTA, ANN, RF, and MAXENT. To prevent artificial inflation of evaluation metrics, pseudo‐absences (*n* = 5000) were not drawn randomly from the entire globe, but were strictly constrained within the biologically defined M areas.

Within the biomod2 framework, individual algorithms were calibrated using cross‐validation. Acknowledging the well‐documented limitations of the Area Under the Curve (AUC) and the True Skill Statistic (TSS) for presence‐only data—particularly their sensitivity to background extent—we restricted their use strictly to internal model building. Specifically, AUC and TSS served solely as mathematical criteria to filter underperforming sub‐models and assign evaluation‐decay weights for the final ensemble assembly. Conversely, all ecological evaluations of the final projection accuracy and spatial transferability were conducted exclusively using the threshold‐independent and presence‐only Continuous Boyce Index (CBI) via the *ecospat* package (Hirzel et al. 2006).

We adopted a two‐step modeling workflow. First, to test the spatial transferability of the native climatic niche, a Native‐calibrated SDM was built exclusively using occurrences and background data from North America. This model was then projected onto the four invaded regions. Model transferability was evaluated using the threshold‐independent Continuous Boyce Index (CBI) via the *ecospat* package, where a CBI approaching 0 indicates poor transferability and potential realized niche divergence. Second, a Global SDM was calibrated by pooling all 4579 occurrences against background points drawn strictly from the comprehensive global M area. Because this global background was delineated by intersecting the entire occurrence dataset (including records outside the five focal regions) with the TEOW shapefile, it provided a broader, yet ecologically justified, accessible extent. This approach successfully prevents the artificial inflation of evaluation metrics caused by global blind sampling, producing a single, robust map that reflects the species' total realized climatic tolerance for risk management. To rigorously evaluate the global model without data circularity, we randomly withheld 30% of the global occurrence records as an independent testing set to calculate the CBI.

### 2.5 Climatic Niche Dynamics and Non‐Analogue Climate Analysis

To explicitly quantify realized niche dynamics without the circularity of using SDM‐derived projections, we directly utilized the true occurrence records within the environmental space framework using the *ecospat* package. We performed a Principal Component Analysis (PCA‐env) calibrated on the combined environmental background (M areas) of the native and respective invaded regions.

To robustly partition the niche and account for non‐analog climates, we decomposed the realized niche into five categories following an extended niche‐dynamics framework based on the COUE approach (Broennimann et al. 2026): Stability (S), Expansion (E; expansion within analog climates), Unfilling (U; unfilling within analog climates), Pioneering (expansion into non‐analog climates absent from the native range), and Abandonment (unfilling of native climates that are completely absent from the invaded range). The proportions of Pioneering (relative to total expansion‐related niche quantity) and Abandonment (relative to total unfilling‐related niche quantity) were calculated to explicitly isolate the influence of environmental availability. Niche equivalency and similarity tests were conducted using 1000 random permutations.

To map these environmental contrasts geographically, we conducted a Multivariate Environmental Similarity Surfaces (MESS) analysis (Elith et al. 2010). Overlaying the true invaded occurrences onto regional MESS maps allowed us to explicitly quantify the proportion of populations established in novel, non‐analog climatic conditions (MESS < 0).

## 3 Results

### 3.1 Native‐Range SDM Performance and Spatial Transferability

The Native‐range ensemble SDM accurately characterized the climatic conditions associated with the native North American distribution. However, when projected globally, spatial transferability varied substantially among invaded regions (Figure 2). The native‐calibrated model showed excellent projection correspondence in Russia–Eastern Europe (CBI = 0.822) and moderate transferability in Western Europe (CBI = 0.623), indicating populations largely occupy climatic conditions analogous to the native range.

**FIGURE 2:**
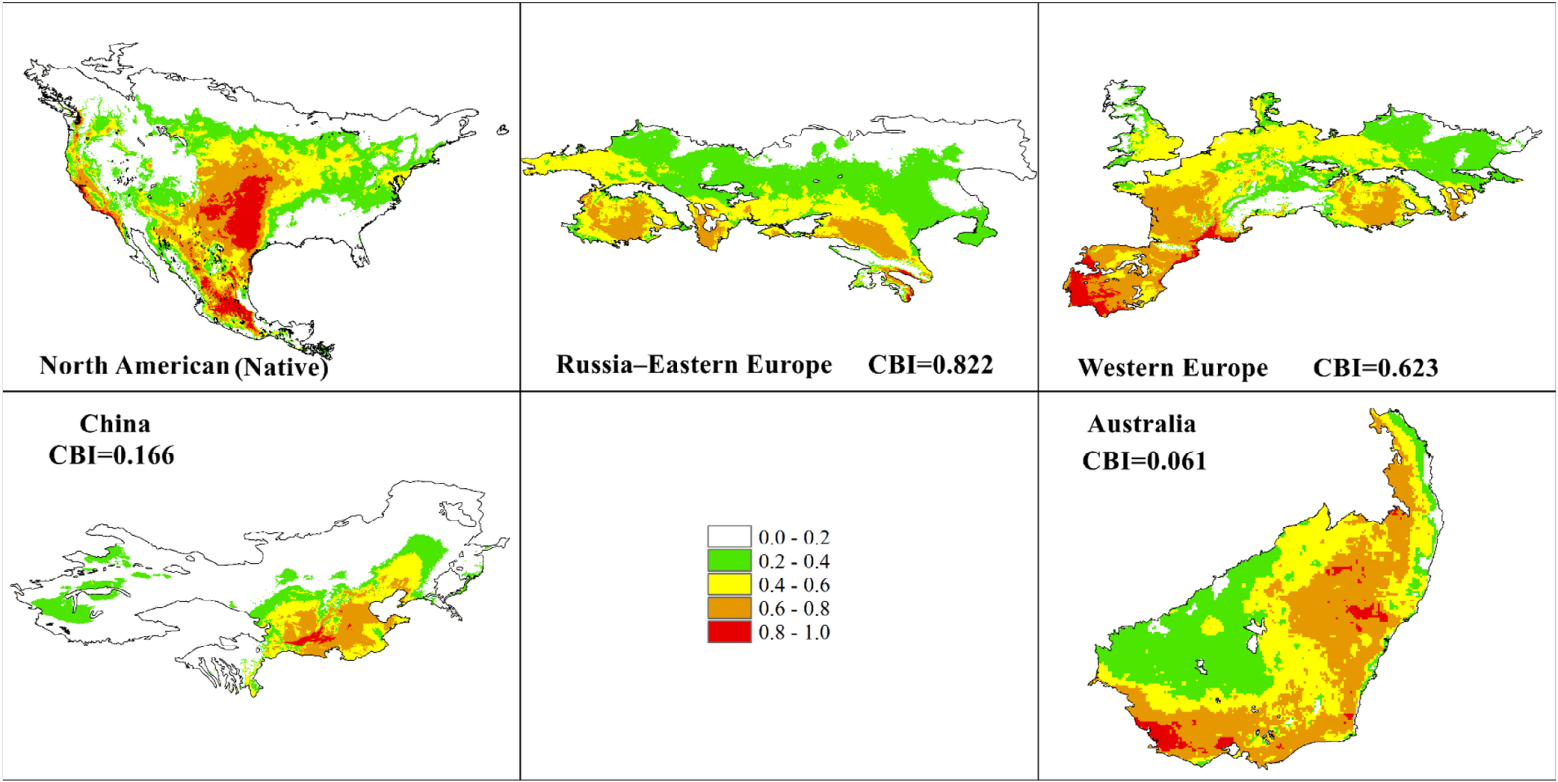
Transferability of the native‐range ensemble SDM across invaded regions of *S. rostratum* . The model was calibrated using North American occurrences and projected onto four invaded regions. Continuous Boyce Index (CBI) values indicate the correspondence between projected climatic suitability and observed occurrence patterns.

In stark contrast, transferability was severely reduced in Australia (CBI = 0.061) and China (CBI = 0.166). While the low transferability in Australia likely reflects the statistical instability of evaluating a highly localized, early‐stage population (*n* = 31) against a vast continent, the failure to project the Chinese distribution indicates a genuine biological shift: extensive regional establishment in environmental spaces unaccounted for by native climatic preferences.

### 3.2 Environmental Drivers Underlying Global Suitability Patterns

Variable contribution analyses from the global SDM revealed that the climatic suitability of *S. rostratum* is temperature‐driven but strictly precipitation‐constrained. The three primary temperature‐related variables—annual mean temperature (bio1; 19.5%), mean temperature of the warmest quarter (bio10; 11.9%), and mean diurnal temperature range (bio2; 11.5%)—cumulatively accounted for 42.9% of the total model contribution. Precipitation of the wettest month (bio13) acted as the secondary, yet critical, limiting factor, contributing 13.4% (Figure 3).

**FIGURE 3:**
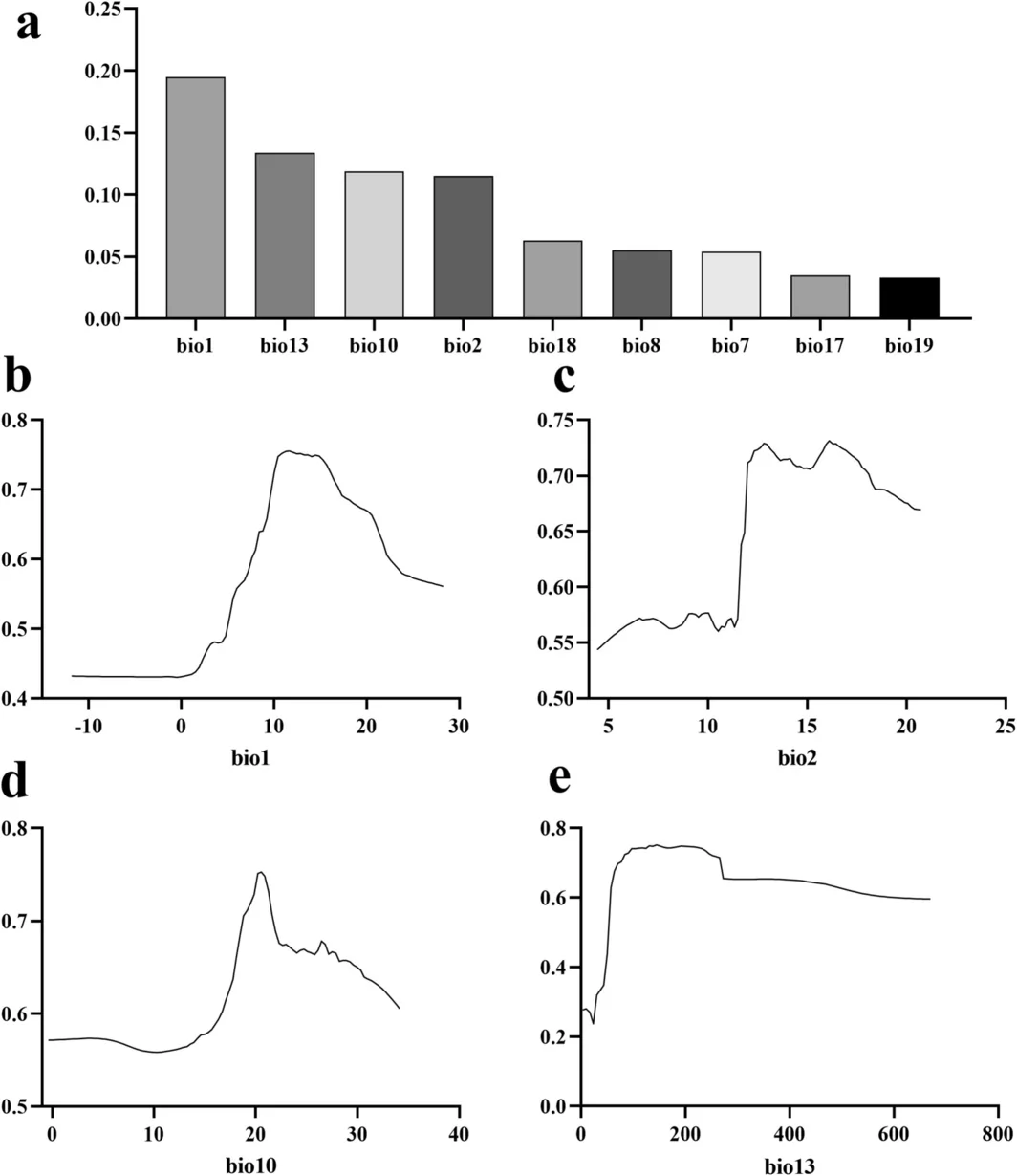
Relative contributions and response relationships of climatic predictors in the global ensemble SDM for *S. rostratum* .

Response curves elucidated the specific climatic thresholds defining optimal suitability. Thermally, suitability peaked at annual mean temperatures (bio1) of 11°C–15°C and summer temperatures (bio10) of 20°C–21°C, followed by gradual declines at higher extremes. Furthermore, the species exhibited a strong preference for pronounced diurnal temperature fluctuations (bio2), with optimal suitability maintained between 13°C and 17°C, characterizing its deep adaptation to continental climates. Regarding moisture availability, suitability increased rapidly at lower precipitation levels but stabilized within a distinct optimal window of 100–250 mm for the wettest month (bio13). Beyond this 250 mm threshold, suitability steadily declined, highlighting that excessive seasonal moisture imposes a strict physiological limit on the species' potential distribution (Figure 3).

### 3.3 Climatic Novelty During Invasion Revealed by MESS Analysis

MESS analysis quantified pronounced regional differences in the non‐analog climates encountered by introduced populations (Figure 4; Table 1). In Australia and Russia–Eastern Europe, introduced populations were located entirely within analogous climatic space (MESS > 0), with 0.00% of occurrences situated in novel climates. Western Europe exhibited minor climatic novelty, with 9.24% of occurrence records located in novel environmental conditions.

**FIGURE 4:**
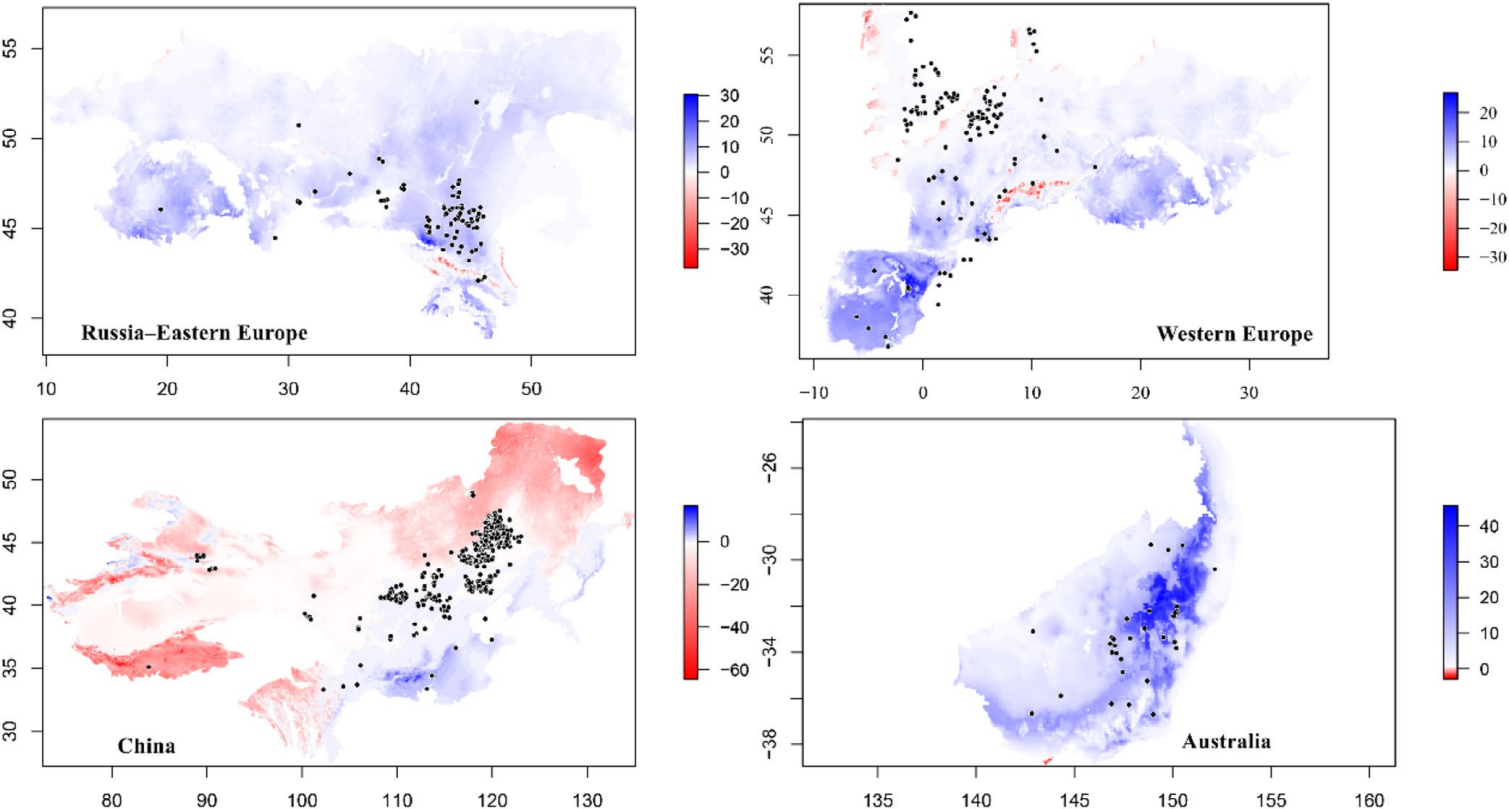
Climatic novelty of invaded populations based on MESS analysis.

**TABLE 1:** Climatic novelty and occurrence patterns across the invaded regions of *S. rostratum* .

Region	Occurrences	Novel climatic space (%)	Analogous climatic space (%)	Occurrences in novel climates (%)	Median occurrence MESS
China	434	74.80	25.20	78.80	−0.29
Russia–Eastern Europe	76	0.86	99.14	0.00	4.55
Australia	31	0.07	99.93	0.00	14.49
Western Europe	126	4.48	95.52	9.24	0.26

Conversely, China presented an extensive exposure to climatic novelty. Approximately 74.80% of the available climatic space in the Chinese M area was non‐analog relative to North America. Consequently, 78.80% of Chinese occurrence records fell strictly within these novel climatic conditions (MESS < 0). This massive colonization of unrepresented climates directly explains the low transferability (CBI = 0.166) of the Native SDM to the Chinese landscape.

### 3.4 Regional Climatic Niche Dynamics Based on PCA‐Env and COUE Analyses

PCA‐env ordinations and the extended COUE decomposition incorporating Pioneering and Abandonment explicitly confirmed that *S. rostratum* displays highly divergent realized niche dynamics across continents (Table 2; Table S3; Figure 5).

**TABLE 2:** Climatic niche overlap and dynamics of *S. rostratum* between the native and introduced ranges.

Region	Schoener's D	Expansion (E)	Stability (S)	Unfilling (U)	Pioneering (% of expansion‐related niche quantity)	Abandonment (% of unfilling‐related niche quantity)	Equivalency *p*	Similarity *p*
China	0.224	0.002	0.998	0.483	48.5	19.9	1.000	0.066
Australia	0.486	0.000	1.000	0.221	—	46.4	0.001	0.017
Western Europe	0.209	0.026	0.974	0.321	0	80.4	0.001	0.131
Russia–Eastern Europe	0.094	0.000	1.000	0.484	—	79.2	0.993	0.010

*Note:* Schoener's D represents climatic niche overlap between native and introduced ranges. Expansion (E), Stability (S), and Unfilling (U) are the standardized niche‐dynamics indices calculated by *ecospat* within the shared analog climatic space. Pioneering represents the proportion of expansion‐related niche quantity occurring in non‐analog environments absent from the native environmental background, calculated as z2_only_NA/(z2_only_NA + z2_only_A). Abandonment represents the proportion of unfilling‐related niche quantity occurring in non‐analog environments absent from the introduced environmental background, calculated as z1_only_NA/(z1_only_NA + z1_only_A). Equivalency and similarity tests were based on 1000 randomizations. An em dash (−) indicates that no expansion‐related niche quantity was detected and therefore the Pioneering proportion was undefined. z1 and z2 represent the occurrence density of the native and introduced populations in the environmental space, respectively. NA denotes non‐analog climates, and A denotes analog climates.

**FIGURE 5:**
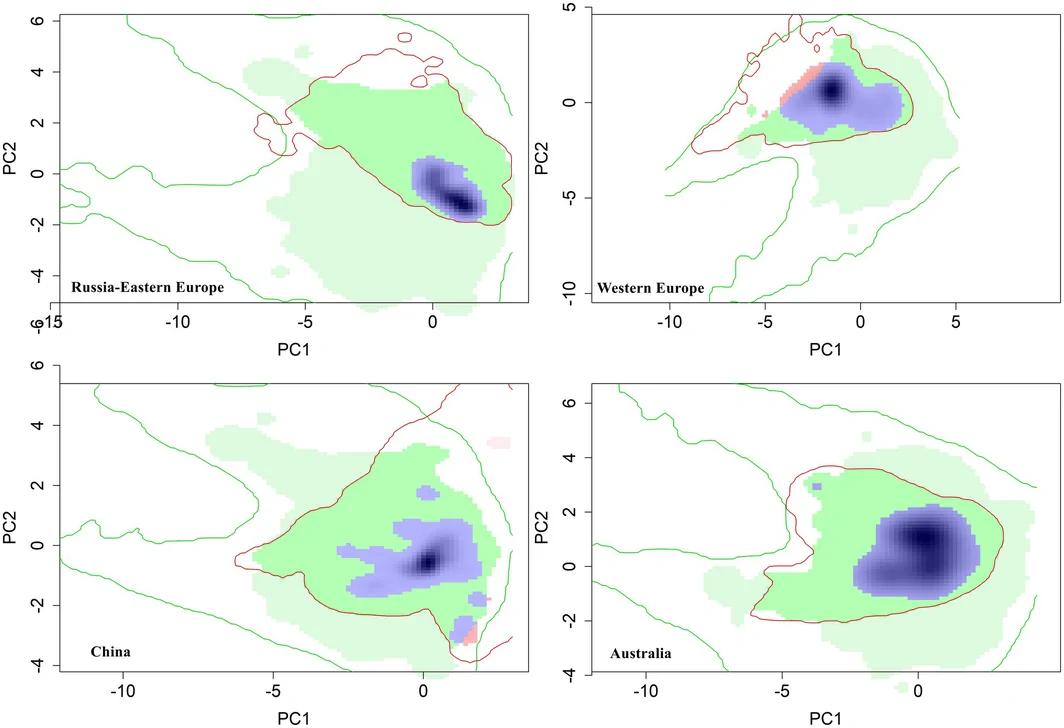
Environmental niche dynamics revealed by PCA‐env and COUE framework.

Australia and Russia–Eastern Europe exhibited strict niche conservatism. Both regions demonstrated complete analog niche stability (S = 1.000) and zero analog expansion (E = 0.000). The observed “unfilling” within shared climates (U = 0.221 and 0.484, respectively) was accompanied by severe environmental limitations: 46.4% and 79.2% of the total unfilling‐related niche quantity (Unfilling + Abandonment) was classified as Abandonment, meaning those native climatic conditions simply do not exist in these invaded territories.

Western European populations displayed moderate analog stability (S = 0.974) and substantial unfilling within shared climates (U = 0.321). However, decomposition revealed that 80.4% of the total unfilling‐related niche quantity is Abandonment. The remaining true unfilling component within shared climate space likely reflects dispersal limitation within the region.

Chinese populations exhibited a highly complex, dual colonization pattern. When evaluated strictly within the subset of climates shared with the native range (the analog space), the species appeared almost perfectly stable (S = 0.998), showing negligible true expansion (E = 0.002). However, this high stability metric does not imply overall niche conservatism. Examining the non‐analog components revealed a massive Pioneer effect. A striking 48.5% of the total expansion‐related niche quantity (Expansion + Pioneering) in China was attributable to non‐analog environments, indicating a substantial contribution of Pioneering to the realized expansion component (Table 2). This demonstrates that while the species perfectly conserves its ancestral niche where available, it possesses the realized capacity to aggressively exploit novel environmental space.

Overall, the PCA‐env and COUE analyses indicate strong regional heterogeneity in climatic niche relationships. Australia and Russia–Eastern Europe were characterized predominantly by climatic stability and environmentally driven abandonment, China showed extensive quantified non‐analog pioneering, and Western Europe exhibited a combination of abandonment and dispersal‐driven unfilling. These regional differences highlight that climatic niche responses during invasion cannot be adequately characterized by a single expansion–conservatism trajectory.

### 3.5 Global Realized‐Niche Model and Worldwide Invasion Risk Assessment

The global ensemble SDM, calibrated using the pooled native and invaded occurrence records against a comprehensive TEOW background, achieved outstanding model performance (independent cross‐validated CBI = 0.999). By integrating the extended realized climatic tolerances observed in China, this model projected extensive regions of high climatic suitability beyond the species' current established distribution (Figure 6). High‐risk areas are intensely concentrated across temperate Eurasia, spanning Europe and Central to East Asia. Additional suitable conditions are prevalent in parts of southern Africa, southern South America, and extensive tracts of Australia, indicating substantial global capacity for further geographical expansion if dispersal pathways remain unmitigated.

**FIGURE 6:**
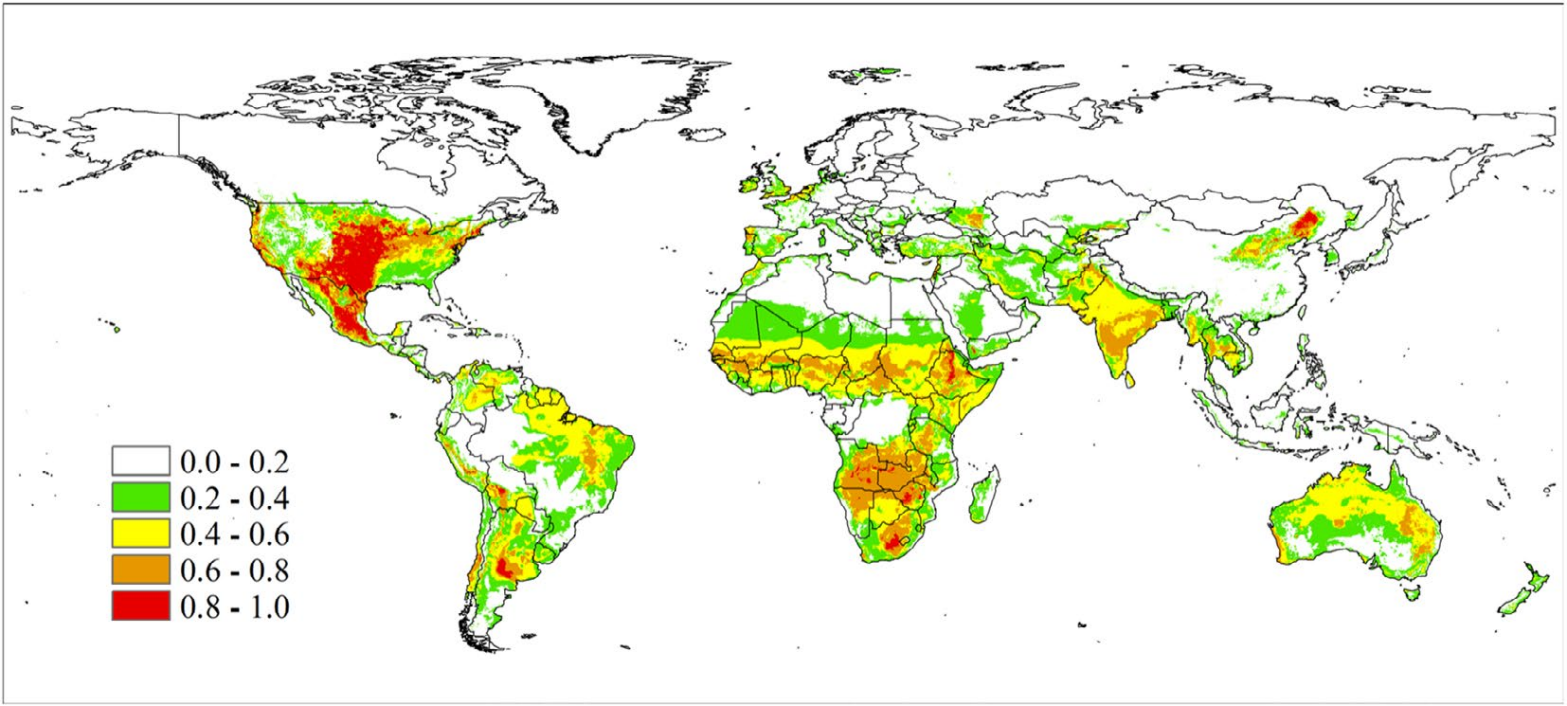
Global projection of climatic suitability for *S. rostratum* based on the realized‐niche ensemble SDM. The suitability score ranges from 0 to 1, with higher values and warmer colors indicating greater climatic suitability and invasion risk.

## 4 Discussion

Our results demonstrate that the global invasion trajectory of *S. rostratum* is not governed by a single monolithic rule but emerges from the complex interplay of regional environmental availability, climatic novelty, and dispersal dynamics. By migrating away from binarized SDM outputs and employing direct environmental‐space comparisons coupled with an extended COUE framework, we bypassed inherent modeling circularities. We found that *S. rostratum* spans an entire spectrum of realized niche dynamics: from strict climatic conservatism in Australia and Russia–Eastern Europe to abandonment‐driven unfilling in Western Europe, to massive non‐analog pioneering in China. This continuum emphasizes that management strategies must dynamically align with the underlying realized niche dynamics rather than relying on static, native‐calibrated risk thresholds.

### 4.1 Climatic Conditions Shaping Climatic Suitability

Macroecological modeling definitively characterized the distribution of *S. rostratum* as temperature‐driven but precipitation‐constrained. The species' reliance on pronounced mean diurnal temperature ranges (bio2) reflects the biogeographical imprint of its North American continental origin (Li et al. 2018; Liu, Zhou, et al. 2022) and its corresponding success in the arid and semi‐arid agro‐pastoral ecotones of Central Asia and Northern China (Yu et al. 2021, 2024). This thermal strategy supports survival in disturbed habitats where intense temperature fluctuations can enhance daytime photosynthesis while reducing nighttime respiration and water loss, optimizing carbon gain and water use efficiency under drought stress (Chen et al. 2012; Guo and Fang 2003; Zhang et al. 2015).

However, although highly drought‐tolerant, its expansion into more humid biomes is strictly barred by excess precipitation (bio13), with virtually no suitable climatic conditions projected in humid subtropical regions. High soil moisture heavily impairs root respiration and vastly increases susceptibility to soil‐borne pathogens, thereby restricting its southward expansion into wet biomes (Jackson and Colmer 2005; Klironomos 2002; Manghwar et al. 2024). Consequently, under future climate change scenarios, thermal warming may relax low‐temperature constraints, promoting potential expansion into high‐latitude or high‐altitude cold regions (Huang, Li, et al. 2024; Wiens and Graham 2005). Conversely, increases in extreme precipitation may restrict its abundance, as high soil moisture elevates root rot risk, potentially creating ephemeral refuges for native communities even in thermally suitable zones (Donat et al. 2016; Jackson and Colmer 2005; Legg 2021; Manghwar et al. 2024; Tabari 2020).

### 4.2 Disentangling Realized Niche Dynamics Across Invaded Regions

The explicit decomposition of non‐analog climate space clarified previously ambiguous niche relationships. While exotic plants frequently exhibit niche conservatism during cross‐continental introductions, *S. rostratum* spans a spectrum from strict conservatism to massive niche expansion, supporting the hypothesis that both mechanisms can coexist during global dispersal (Atwater et al. 2018; Petitpierre et al. 2012).

In Australia and Russia–Eastern Europe, the invasion is characterized by strict realized climatic conservatism. Although the native SDM yielded a low predictive score (CBI) for Australia, this is a known statistical artifact when evaluating models against extremely small and highly clustered populations (*n* = 31). Our MESS analyses definitively resolved this, confirming that introduced populations are entirely restricted to analog climatic conditions, and the extended COUE decomposition showed zero active expansion. Crucially, the apparent unfilling in these regions was largely unmasked as Abandonment (46.4% and 79.2%, respectively). The species is not actively avoiding suitable native climates; those specific climates simply do not exist within the respective invaded biogeographic extents. In Australia, this conservatism is likely maintained by limited genetic variation due to founder effects, combined with geographic barriers that prevent access to other potentially suitable environments (Gallagher et al. 2010). Similarly, distribution in Russia–Eastern Europe is currently constrained by dispersal limitation rather than physiological boundaries (Early and Sax 2014).

Western Europe exhibits a related pattern characterized by high Abandonment (80.4%), meaning the bulk of the unfilling is environmentally driven. However, the residual true unfilling within analog climates suggests genuine dispersal limitation; suitable European climates exist that have not yet been reached by the current invasion front, supporting the apparent niche shift hypothesis (Broennimann et al. 2012; Guisan et al. 2014).

In stark contrast, populations in China exhibit extensive pioneering into non‐analog climates. The Native‐calibrated SDM failed entirely to project the Chinese distribution (CBI = 0.166). The MESS analysis unequivocally demonstrated that 78.8% of occurrences fall within novel climates, and the raw COUE decomposition verified that a striking 48.5% of the expansion‐related niche quantity represents true pioneering into environments utterly absent from the North American baseline. Interestingly, the standardized stability metric (S = 0.998) shows that where native‐like climates do exist in China, the species strictly conserves its ancestral niche. This realized niche expansion into novel environments may be driven by high phenotypic plasticity, which buffers against environmental stress (Davidson et al. 2011). Alternatively, it may result from ecological release; the absence of specialist herbivores and pathogens could allow the species to allocate more resources to abiotic stress tolerance, occupying climatic spaces from which it is competitively excluded in its native range (Keane and Crawley 2002). Furthermore, multiple introductions and genetic admixture may have provided the standing genetic variation necessary to facilitate such rapid expansion (Lombaert et al. 2010; Zhao and Lou 2017).

### 4.3 Implications for Management and Biodiversity Conservation

The niche dynamic framework developed here provides an important extension to traditional static invasion‐risk assessments. While translating model inaccuracies into absolute spatial areas (e.g., hectares of underestimated risk) is methodologically unsound due to unreliable extrapolation in non‐analog climates, integrating CBI and MESS provides a quantitative framework for relative risk ranking. Our results demonstrate that models calibrated solely on native‐range data systematically underestimate invasion potential in regions where introduced populations pioneer novel climatic conditions (e.g., regional CBI < 0.4 and > 50% of occurrences with MESS < 0, as in China). Conversely, these models retain relatively high transferability in regions characterized by strictly analog environments and climatic conservatism (e.g., regional CBI > 0.8 and no occurrences with MESS < 0, as in Russia–Eastern Europe) (Broennimann et al. 2007). Thus, incorporating occurrence records globally generates a comprehensive realized‐niche model (CBI = 0.999) that captures a more accurate operational risk map for policymakers.

Management interventions should be tailored to regional niche profiles. In strictly conservative regions (e.g., Australia), populations remain highly localized. This isolation provides a highly cost‐effective window for eradication (Crooks et al. 1999; Panetta 2009). Strict biosecurity is required to prevent new genetic introductions that could trigger renewed expansion. In unfilling regions (e.g., Europe), where distribution changes are primarily driven by dispersal limitation, Early Detection and Rapid Response (EDRR) systems are essential. Because propagule pressure strongly influences invasion success, surveillance should prioritize ports and logistics hubs to disrupt introduction pathways (Lockwood et al. 2005), potentially integrating remote sensing to identify large‐scale soil disturbances that often precede establishment (Rejmánek and Pitcairn 2003).

In regions experiencing active non‐analog pioneering (e.g., China), management must transcend static climatic thresholds and account for high phenotypic plasticity and potential local adaptation (Colautti and Lau 2016). Because long‐distance spread is strongly associated with agricultural machinery and forage transport, pathway‐based quarantine is critical (Hulme 2009). Ecological restoration strategies should exploit the species' physiological limitations. Establishing dense, multi‐layered vegetation to impose light limitation is likely more effective than planting single native grasses, particularly under elevated nitrogen deposition which enhances the invader's competitiveness (Funk et al. 2008; Sun et al. 2023).

### 4.4 Limitations and Future Directions

While this study leverages robust occurrence‐based environmental comparisons, it exclusively measures the realized niche, not the fundamental niche. Correlative niche models cannot disentangle the precise mechanisms driving the massive climatic pioneering observed in China. The colonization of novel cold and arid environments could reflect high pre‐existing phenotypic plasticity, ecological release from native specialist herbivores, multiple introductions generating high standing genetic variation (founder admixture), or true rapid evolutionary adaptation. Future research must integrate multidisciplinary approaches, pairing population genomic analyses with reciprocal common garden experiments, to definitively distinguish phenotypic plasticity from fundamental niche evolution during global invasion.

## 5 Conclusions

This study clarifies the cross‐continental colonization dynamics of *S. rostratum* , yielding three central insights. First, its macroecological spread is fundamentally constrained by a “temperature‐driven, precipitation‐constrained” mechanism. Second, the species utilizes a highly heterogeneous invasion strategy: it strictly conserves its climatic niche and experiences environmentally‐driven abandonment in regions like Australia and Europe, yet aggressively pioneers non‐analog, novel climates in China (accounting for 48.5% of its expansion‐related niche quantity). Third, because species can dynamically colonize novel environments during spread, static risk projections using only native data routinely underestimate global invasion risk. Employing a global ensemble SDM that captures these expanded realized tolerances provides a more rigorous, region‐specific baseline for managing invasive alien species under shifting climates.

## Author Contributions

**Yabin Liu:** investigation (equal), visualization (equal), writing – original draft (equal). **Weitian Meng:** investigation (equal), software (equal). **Lizhu Guo:** investigation (equal), software (equal). **Yuyu Li:** investigation (equal), methodology (equal). **Rui Wang:** investigation (equal), methodology (equal), software (equal). **Chuan Wang:** investigation (equal), methodology (equal), project administration (equal). **Yu Ji:** investigation (equal), methodology (equal). **Tian Tian:** investigation (equal), methodology (equal). **Lifen Hao:** conceptualization (equal), methodology (equal), writing – review and editing (equal). **Kejian Lin:** conceptualization (equal), funding acquisition (equal), methodology (equal).

## Funding

This work was supported by the Ministry of Science and Technology of the People's Republic of China (National Key Research and Development Program of China, 2024YFC2607700), the Ministry of Agriculture and Rural Affairs of the People's Republic of China (China Forage and Grass Research System CARS, CARS‐33), and the Department of Science and Technology of Inner Mongolia Autonomous Region (Inner Mongolia Autonomous Region Science & Technology Plan Project, 2025YFHH0163).

## Conflicts of Interest

The authors declare no conflicts of interest.

## Supporting information


**Data S1:** Supplementary Information.

**Figure S1:** Comparison of frequency histograms of MESS values between the native range and introduced ranges of *S. rostratum* . Using North American native climate as reference, each histogram shows MESS values (x‑axis) versus the frequency of 5‑arc‑minute grid cells (y‑axis); positive values indicate climatic similarity, negative values indicate dissimilarity, and the red dashed line marks the zero threshold. The side‑by‑side layout allows visual assessment of climatic niche deviation in each introduced region relative to the native range.

**Table S1:** Combination of climate variables in species distribution models undergoing testing.
**Table S2:** Accuracy evaluation of 12 combinations.
**Table S3:** Quantitative decomposition of climatic niche‐dynamics categories returned by ecospat.

## Data Availability

The data that support the findings of this study are openly available in the Figure S1; Table S1–S3 of this article. The original occurrence records were obtained from public databases (GBIF, iNaturalist, CABI, CalFlora, Atlas of Living Australia, and SEINet). The final curated occurrence datasets (.csv), accessible area shapefiles (.shp), and custom analysis R scripts (.R) used in this study are provided as Data S1.

## References

[ece374662-bib-0001] Aiello‐Lammens, M. E. , R. A. Boria , A. Radosavljevic , B. Vilela , and R. P. Anderson . 2015. “spThin: An R Package for Spatial Thinning of Species Occurrence Records for Use in Ecological Niche Models.” Ecography 38, no. 5: 541–545. 10.1111/ecog.01132.

[ece374662-bib-0002] Atwater, D. Z. , C. Ervine , and J. N. Barney . 2018. “Climatic Niche Shifts Are Common in Introduced Plants.” Nature Ecology & Evolution 2, no. 1: 34–43. 10.1038/s41559-017-0396-z. 29203919

[ece374662-bib-0003] Biancolini, D. , O. Broennimann , A. Guisan , and C. Rondinini . 2026. “Global Patterns of Niche Changes in Alien Mammals: Potential Drivers and Significance for Invasion Projections.” Global Change Biology 32, no. 3: e70755. 10.1111/gcb.70755. PMC13004023 41860462

[ece374662-bib-0004] Broennimann, O. , F. Collart , and A. Guisan . 2026. “The Ecospat R Package: A Collection of Pre‐, Core‐, and Post‐Modeling Tools to Investigate Species Niches and Distributions.” In R Coding for Ecology, vol. 67–98. Springer Nature Switzerland. 10.1007/978-3-031-99665-8_3.

[ece374662-bib-0005] Broennimann, O. , M. C. Fitzpatrick , P. B. Pearman , et al. 2012. “Measuring Ecological Niche Overlap From Occurrence and Spatial Environmental Data.” Global Ecology and Biogeography 21, no. 4: 481–497. 10.1111/j.1466-8238.2011.00698.x.

[ece374662-bib-0006] Broennimann, O. , U. A. Treier , H. Müller‐Schärer , W. Thuiller , A. T. Peterson , and A. Guisan . 2007. “Evidence of Climatic Niche Shift During Biological Invasion.” Ecology Letters 10, no. 8: 701–709. 10.1111/j.1461-0248.2007.01060.x. 17594425

[ece374662-bib-0007] Chen, T. , W. Liu , C. Zhang , and J. Wang . 2012. “Effects of *Solidago canadensis* Invasion on Dynamics of Native Plant Communities and Their Mechanisms.” Chinese Journal of Plant Ecology 36, no. 3: 253–261. 10.3724/SP.J.1258.2012.00253.

[ece374662-bib-0008] Christina, M. , F. Limbada , and A. Atlan . 2020. “Climatic Niche Shift of an Invasive Shrub ( *Ulex europaeus* ): A Global Scale Comparison in Native and Introduced Regions.” Journal of Plant Ecology 13, no. 1: 42–50. 10.1093/jpe/rtz041.

[ece374662-bib-0009] Colautti, R. I. , and J. A. Lau . 2016. “Contemporary Evolution During Invasion: Evidence for Differentiation, Natural Selection, and Local Adaptation.” In Invasion Genetics, 101–121. Wiley. 10.1002/9781119072799.ch6. 25891044

[ece374662-bib-0010] Crooks, J. A. , M. E. Soulé , and O. T. Sandlund . 1999. “Lag Times in Population Explosions of Invasive Species: Causes and Implications.” In Invasive Species and Biodiversity Management, vol. 24, 103–125. Springer. 10.1007/978-94-011-4523-7_7.

[ece374662-bib-0011] Davidson, A. M. , M. Jennions , and A. B. Nicotra . 2011. “Do Invasive Species Show Higher Phenotypic Plasticity Than Native Species and, if So, Is It Adaptive? A Meta‐Analysis.” Ecology Letters 14, no. 4: 419–431. 10.1111/j.1461-0248.2011.01596.x. 21314880

[ece374662-bib-0012] Deslippe, J. R. , and J. A. Veenendaal . 2025. “Plant Invasions in a Changing Climate: Reshaping Communities, Ecosystem Functions, and Services.” Annual Review of Ecology, Evolution, and Systematics 56: 571–596. 10.1146/annurev-ecolsys-102723-040443.

[ece374662-bib-0013] Di Cola, V. , O. Broennimann , B. Petitpierre , et al. 2017. “Ecospat: An R Package to Support Spatial Analyses and Modeling of Species Niches and Distributions.” Ecography 40, no. 6: 774–787. 10.1111/ecog.02671.

[ece374662-bib-0014] Dlugosch, K. M. , and I. M. Parker . 2008. “Founding Events in Species Invasions: Genetic Variation, Adaptive Evolution, and the Role of Multiple Introductions.” Molecular Ecology 17, no. 1: 431–449. 10.1111/j.1365-294X.2007.03538.x. 17908213

[ece374662-bib-0015] Donat, M. G. , A. L. Lowry , L. V. Alexander , P. A. O'Gorman , and N. Maher . 2016. “More Extreme Precipitation in the World's Dry and Wet Regions.” Nature Climate Change 6, no. 5: 508–513. 10.1038/nclimate2941.

[ece374662-bib-0016] Early, R. , B. A. Bradley , J. S. Dukes , et al. 2016. “Global Threats From Invasive Alien Species in the Twenty‐First Century and National Response Capacities.” Nature Communications 7, no. 1: 1–9. 10.1038/ncomms12485. PMC4996970 27549569

[ece374662-bib-0017] Early, R. , and D. F. Sax . 2014. “Climatic Niche Shifts Between Species' Native and Naturalized Ranges Raise Concern for Ecological Forecasts During Invasions and Climate Change.” Global Ecology and Biogeography 23, no. 12: 1356–1365. 10.1111/geb.12208.

[ece374662-bib-0018] Elith, J. , M. Kearney , and S. Phillips . 2010. “The Art of Modelling Range‐Shifting Species.” Methods in Ecology and Evolution 1, no. 4: 330–342. 10.1111/j.2041-210X.2010.00036.x.

[ece374662-bib-0019] Feijó, A. , D. Y. Ge , Z. X. Wen , J. L. Cheng , L. Xia , and Q. S. Yang . 2021. “Exploring GBIF Database and Extracting Climate Data From Georeferenced Localities With R Software.” Bio‐101: e1010609. 10.21769/BioProtoc.1010609.

[ece374662-bib-0020] Flickinger, H. D. , and J. S. Dukes . 2024. “A Review of Theory: Comparing Invasion Ecology and Climate Change‐Induced Range Shifting.” Global Change Biology 30, no. 12: e17612. 10.1111/gcb.17612. PMC11647056 39676232

[ece374662-bib-0021] Funk, J. L. , E. E. Cleland , K. N. Suding , and E. S. Zavaleta . 2008. “Restoration Through Reassembly: Plant Traits and Invasion Resistance.” Trends in Ecology & Evolution 23, no. 12: 695–703. 10.1016/j.tree.2008.07.013. 18951652

[ece374662-bib-0022] Gallagher, R. V. , L. J. Beaumont , L. Hughes , and M. R. Leishman . 2010. “Evidence for Climatic Niche and Biome Shifts Between Native and Novel Ranges in Plant Species Introduced to Australia.” Journal of Ecology 98, no. 4: 790–799. 10.1111/j.1365-2745.2010.01677.x.

[ece374662-bib-0023] Guisan, A. , B. Petitpierre , O. Broennimann , C. Daehler , and C. Kueffer . 2014. “Unifying Niche Shift Studies: Insights From Biological Invasions.” Trends in Ecology & Evolution 29, no. 5: 260–269. 10.1016/j.tree.2014.02.009. 24656621

[ece374662-bib-0024] Guo, S. , and F. Fang . 2003. “Physiological Adaptation of the Invasive Plant *Solidago canadensis* to Environments.” Chinese Journal of Plant Ecology 27, no. 1: 47–52. 10.17521/cjpe.2003.0007.

[ece374662-bib-0025] Hellmann, J. J. , J. E. Byers , B. G. Bierwagen , and J. S. Dukes . 2008. “Five Potential Consequences of Climate Change for Invasive Species.” Conservation Biology 22, no. 3: 534–543. 10.1111/j.1523-1739.2008.00951.x. 18577082

[ece374662-bib-0026] Hirzel, A. H. , G. Le Lay , V. Helfer , C. Randin , and A. Guisan . 2006. “Evaluating the Ability of Habitat Suitability Models to Predict Species Presences.” Ecological Modelling, Predicting Species Distributions 199, no. 2: 142–152. 10.1016/j.ecolmodel.2006.05.017.

[ece374662-bib-0027] Huang, T. , T. Yang , K. Wang , and W. Huang . 2024. “Assessing the Current and Future Potential Distribution of *Solanum rostratum* Dunal in China Using Multisource Remote Sensing Data and Principal Component Analysis.” Remote Sensing 16, no. 2: 271. 10.3390/rs16020271.

[ece374662-bib-0028] Huang, Y. , T. Li , W. Chen , et al. 2024. “Analysis of the Distribution Pattern of Phenacoccus Manihoti in China Under Climate Change Based on the Biomod2 Model.” Biology 13, no. 7: 538. 10.3390/biology13070538. PMC11274023 39056731

[ece374662-bib-0029] Hulme, P. E. 2009. “Trade, Transport and Trouble: Managing Invasive Species Pathways in an Era of Globalization.” Journal of Applied Ecology 46, no. 1: 10–18. 10.1111/j.1365-2664.2008.01600.x.

[ece374662-bib-0030] Hulme, P. E. 2021. “Unwelcome Exchange: International Trade as a Direct and Indirect Driver of Biological Invasions Worldwide.” One Earth 4, no. 5: 666–679. 10.1016/j.oneear.2021.04.015.

[ece374662-bib-0031] Izzo, V. M. , N. Mercer , J. Armstrong , and Y. H. Chen . 2014. “Variation in Host Usage Among Geographic Populations of *Leptinotarsa decemlineata* , the Colorado Potato Beetle.” Journal of Pest Science 87, no. 4: 597–608. 10.1007/s10340-014-0578-2.

[ece374662-bib-0032] Jackson, M. B. , and T. D. Colmer . 2005. “Response and Adaptation by Plants to Flooding Stress.” Annals of Botany 96, no. 4: 501–505. 10.1093/aob/mci205. PMC4247020 16217870

[ece374662-bib-0033] Jaureguiberry, P. , N. Titeux , M. Wiemers , et al. 2022. “The Direct Drivers of Recent Global Anthropogenic Biodiversity Loss.” Science Advances 8, no. 45: eabm9982. 10.1126/sciadv.abm9982. PMC9645725 36351024

[ece374662-bib-0034] Keane, R. M. , and M. J. Crawley . 2002. “Exotic Plant Invasions and the Enemy Release Hypothesis.” Trends in Ecology & Evolution 17, no. 4: 164–170. 10.1016/S0169-5347(02)02499-0.

[ece374662-bib-0035] Klironomos, J. N. 2002. “Feedback With Soil Biota Contributes to Plant Rarity and Invasiveness in Communities.” Nature 417, no. 6884: 67–70. 10.1038/417067a. 11986666

[ece374662-bib-0036] Legg, S. 2021. “IPCC, 2021: Climate Change 2021‐The Physical Science Basis.” Interaction 49, no. 4: 44–45. 10.3316/informit.315096509383738.

[ece374662-bib-0037] Li, Z. , Y. Wang , and W. Li . 2018. “Spatiotemporal Variation of Evaporation and Transpiration From 1982‐2012 in the Arid Valley Region in Northwest China.” Journal of Irrigation and Drainage 37, no. 7: 120–128. 10.13522/j.cnki.ggps.2017.0679.

[ece374662-bib-0038] Lin, Y. , and D. Y. Tan . 2007. “The Potential and Exotic Invasive Plant: *Solanum rostratum* .” Acta Phytotaxonomica Sinica 5: 675–685. 10.1360/aps07010.

[ece374662-bib-0039] Liu, C. , C. Wolter , F. Courchamp , N. Roura‐Pascual , and J. M. Jeschke . 2022. “Biological Invasions Reveal How Niche Change Affects the Transferability of Species Distribution Models.” Ecology 103, no. 8: e3719. 10.1002/ecy.3719. 35388469

[ece374662-bib-0040] Liu, C. , J. Zhou , X. Ma , Z. Xie , and G. Yang . 2022. “Research Progress on Forage Utilization of Shrub Plants in Arid Area of Northwest China.” Journal of Agricultural Science and Technology 24, no. 3: 28–39. 10.13304/j.nykjdb.2021.0074.

[ece374662-bib-0041] Lockwood, J. L. , P. Cassey , and T. Blackburn . 2005. “The Role of Propagule Pressure in Explaining Species Invasions.” Trends in Ecology & Evolution 20, no. 5: 223–228. 10.1016/j.tree.2005.02.004. 16701373

[ece374662-bib-0042] Lombaert, E. , T. Guillemaud , J.‐M. Cornuet , T. Malausa , B. Facon , and A. Estoup . 2010. “Bridgehead Effect in the Worldwide Invasion of the Biocontrol Harlequin Ladybird.” PLoS One 5, no. 3: e9743. 10.1371/journal.pone.0009743. PMC2840033 20305822

[ece374662-bib-0043] Mancini, G. , M. Di Marco , M. Carboni , P. Cerretti , L. Maiorano , and L. Santini . 2025. “On the Importance of Expert‐Informed Variable Selection in Species Distribution Modelling.” Journal of Biogeography 52, no. 11: e70037. 10.1111/jbi.70037.

[ece374662-bib-0044] Manghwar, H. , A. Hussain , I. Alam , M. A. Khoso , Q. Ali , and F. Liu . 2024. “Waterlogging Stress in Plants: Unraveling the Mechanisms and Impacts on Growth, Development, and Productivity.” Environmental and Experimental Botany 224: 105824. 10.1016/j.envexpbot.2024.105824.

[ece374662-bib-0045] Matzrafi, M. , J. Abu‐Nassar , C. Klap , M. Shtarkman , E. Smith , and A. Dombrovsky . 2023. “Solanum Elaeagnifolium and *S. rostratum* as Potential Hosts of the Tomato Brown Rugose Fruit Virus.” PLoS One 18, no. 3: e0282441. 10.1371/journal.pone.0282441. PMC9977001 36857395

[ece374662-bib-0046] Melton, A. E. , M. H. Clinton , D. N. Wasoff , et al. 2022. “Climatic Niche Comparisons of Eastern North American and Eastern Asian Disjunct Plant Genera.” Global Ecology and Biogeography 31, no. 7: 1290–1302. 10.1111/geb.13504.

[ece374662-bib-0047] Olson, D. M. , E. Dinerstein , E. D. Wikramanayake , et al. 2001. “Terrestrial Ecoregions of the World: A New Map of Life on Earth: A New Global Map of Terrestrial Ecoregions Provides an Innovative Tool for Conserving Biodiversity.” Bioscience 51, no. 11: 933–938.

[ece374662-bib-0048] Panetta, F. D. 2009. “Weed Eradication—An Economic Perspective.” Invasive Plant Science and Management 2, no. 4: 360–368. 10.1614/IPSM-09-003.1.

[ece374662-bib-0049] Petitpierre, B. , C. Kueffer , O. Broennimann , C. Randin , C. Daehler , and A. Guisan . 2012. “Climatic Niche Shifts Are Rare Among Terrestrial Plant Invaders.” Science 335, no. 6074: 1344–1348. 10.1126/science.1215933. 22422981

[ece374662-bib-0050] Rejmánek, M. , and M. J. Pitcairn . 2003. “When Is Eradication of Exotic Pest Plants a Realistic Goal?” In Turning the Tide: The Eradication of Invasive Species: Proceedings of the International Conference on Eradication of Island Invasives, edited by M. N. C. C. R. Veitch , 249–253. IUCN‐The World Conservation Union.

[ece374662-bib-0051] Rojas‐Soto, O. , J. S. Forero‐Rodríguez , A. Galindo‐Cruz , et al. 2024. “Calibration Areas in Ecological Niche and Species Distribution Modelling: Unravelling Approaches and Concepts.” Journal of Biogeography 51, no. 8: 1416–1428. 10.1111/jbi.14834.

[ece374662-bib-0052] Rubenstein, M. A. , S. R. Weiskopf , R. Bertrand , et al. 2023. “Climate Change and the Global Redistribution of Biodiversity: Substantial Variation in Empirical Support for Expected Range Shifts.” Environmental Evidence 12, no. 1: 1–21. 10.1186/s13750-023-00296-0. PMC11378804 39294691

[ece374662-bib-0053] Sun, J. K. , M. C. Liu , K. Q. Tang , et al. 2023. “Advantages of Growth and Competitive Ability of the Invasive Plant *Solanum rostratum* Over Two Co‐Occurring Natives and the Effects of Nitrogen Levels and Forms.” Frontiers in Plant Science 14: 1169317. 10.3389/fpls.2023.1169317. PMC10151799 37143880

[ece374662-bib-0054] Tabari, H. 2020. “Climate Change Impact on Flood and Extreme Precipitation Increases With Water Availability.” Scientific Reports 10, no. 1: 13768. 10.1038/s41598-020-70816-2. PMC7426818 32792563

[ece374662-bib-0055] Turbelin, A. J. , R. N. Cuthbert , F. Essl , P. J. Haubrock , A. Ricciardi , and F. Courchamp . 2023. “Biological Invasions Are as Costly as Natural Hazards.” Perspectives in Ecology and Conservation 21, no. 2: 143–150. 10.1016/j.pecon.2023.03.002.

[ece374662-bib-0056] Wallingford, P. D. , T. L. Morelli , J. M. Allen , et al. 2020. “Adjusting the Lens of Invasion Biology to Focus on the Impacts of Climate‐Driven Range Shifts.” Nature Climate Change 10, no. 5: 398–405. 10.1038/s41558-020-0768-2.

[ece374662-bib-0057] Wang, J. , J. Zhao , L. Jiang , X. Han , and Y. Zhu . 2025. “Predicting the Potential Suitable Habitat of *Solanum rostratum* in China Using the Biomod2 Ensemble Modeling Framework.” Plants 14, no. 17: 2779. 10.3390/plants14172779. PMC12430113 40941945

[ece374662-bib-0058] Wiens, J. J. , and C. H. Graham . 2005. “Niche Conservatism: Integrating Evolution, Ecology, and Conservation Biology.” Annual Review of Ecology, Evolution, and Systematics 36: 519–539. 10.1146/annurev.ecolsys.36.102803.095431.

[ece374662-bib-0059] Yu, H. , R. Zhang , W. Huang , et al. 2024. “Seed Traits and Germination of Invasive Plant *Solanum rostratum* (Solanaceae) in the Arid Zone of Northern China Indicate Invasion Patterns.” Plants 13, no. 23: 3287. 10.3390/plants13233287. PMC11644733 39683078

[ece374662-bib-0060] Yu, H. , X. Zhao , W. Huang , J. Zhan , and Y. He . 2021. “Drought Stress Influences the Growth and Physiological Characteristics of *Solanum rostratum* Dunal Seedlings From Different Geographical Populations in China.” Frontiers in Plant Science 12: 733268. 10.3389/fpls.2021.733268. PMC8637895 34868115

[ece374662-bib-0061] Zhang, H. , M. Zhu , and G. Li . 2015. “Factors Influencing the Nonrandom Abscission of *Solidago canadensis* Seeds.” Chinese Journal of Plant Ecology 39, no. 3: 258–263. 10.17521/cjpe.2015.0025.

[ece374662-bib-0062] Zhao, J. , and A. Lou . 2017. “Genetic Diversity and Population Structure of the Invasive Plant *Solanum rostratum* in China.” Russian Journal of Ecology 48, no. 2: 134–142. 10.1134/S1067413617220039.

[ece374662-bib-0063] Zhao, J. , L. Solís‐Montero , A. Lou , and M. Vallejo‐Marín . 2013. “Population Structure and Genetic Diversity of Native and Invasive Populations of *Solanum rostratum* (Solanaceae).” PLoS One 8, no. 11: e79807. 10.1371/journal.pone.0079807. PMC3818217 24224008

[ece374662-bib-0064] Zizka, A. , D. Silvestro , T. Andermann , et al. 2019. “CoordinateCleaner: Standardized Cleaning of Occurrence Records From Biological Collection Databases.” Methods in Ecology and Evolution 10, no. 5: 744–751. 10.1111/2041-210X.13152.

