# Supplementary material for: Global Potential Distribution and Climatic Niche Dynamics of the Invasive Alien Weed Solanum rostratum Dunal
Source: Ecol Evol. 2026 Nov 9;16(11):e74662. doi: 10.1002/ece3.74662 (PMC13747726; doi:10.1002/ece3.74662)
Supplement: Supplementary file 1 [file ECE3-16-e74662-s001.docx]

Fig. S1 Comparison of frequency histograms of MESS values between the native range and introduced ranges of *S. rostratum*. Using North American native climate as reference, each histogram shows MESS values (x‑axis) versus the frequency of 5‑arc‑minute grid cells (y‑axis); positive values indicate climatic similarity, negative values indicate dissimilarity, and the red dashed line marks the zero threshold. The side‑by‑side layout allows visual assessment of climatic niche deviation in each introduced region relative to the native range.
